# Future horizons in diabetes: integrating AI and personalized care

**DOI:** 10.3389/fendo.2025.1583227

**Published:** 2025-03-27

**Authors:** Kaiqi Zhang, Yun Qi, Wenjun Wang, Xinyi Tian, Jiahui Wang, Lili Xu, Xu Zhai

**Affiliations:** ^1^ Wangjing Hospital, China Academy of Chinese Medical Sciences, Beijing, China; ^2^ Rehabilitation Department, Naval Qingdao Special Service Rehabilitation Center, Qingdao, China; ^3^ Xiyuan Hospital, China Academy of Chinese Medical Sciences, Beijing, China; ^4^ School of Acupuncture and Tuina, Shandong University of Traditional Chinese Medicine, Jinan, China; ^5^ School of Health, Shandong University of Traditional Chinese Medicine, Jinan, China; ^6^ Graduate school, China Academy of Chinese Medical Sciences, Beijing, China

**Keywords:** diabetes blood glucose, glucose variability, time in range, continuous glucose monitoring, taVNS, artificial intelligence

## Abstract

Diabetes is a global health crisis with rising incidence, mortality, and economic burden. Traditional markers like HbA1c are insufficient for capturing short-term glycemic fluctuations, leading to the need for more precise metrics such as Glucose Variability (GV) and Time in Range (TIR). Continuous Glucose Monitoring (CGM) and AI integration offer real-time data analytics and personalized treatment plans, enhancing glycemic control and reducing complications. The combination of transcutaneous auricular vagus nerve stimulation (taVNS) with artificial Intelligence (AI) further optimizes glucose regulation and addresses comorbidities. Empowering patients through AI-driven self-management and community support is crucial for sustainable improvements. Future horizons in diabetes care must focus on overcoming challenges in data privacy, algorithmic bias, device interoperability, and equity in AI-driven care while integrating these innovations into healthcare systems to improve patient outcomes and quality of life.

## Introduction

**Figure 1:**
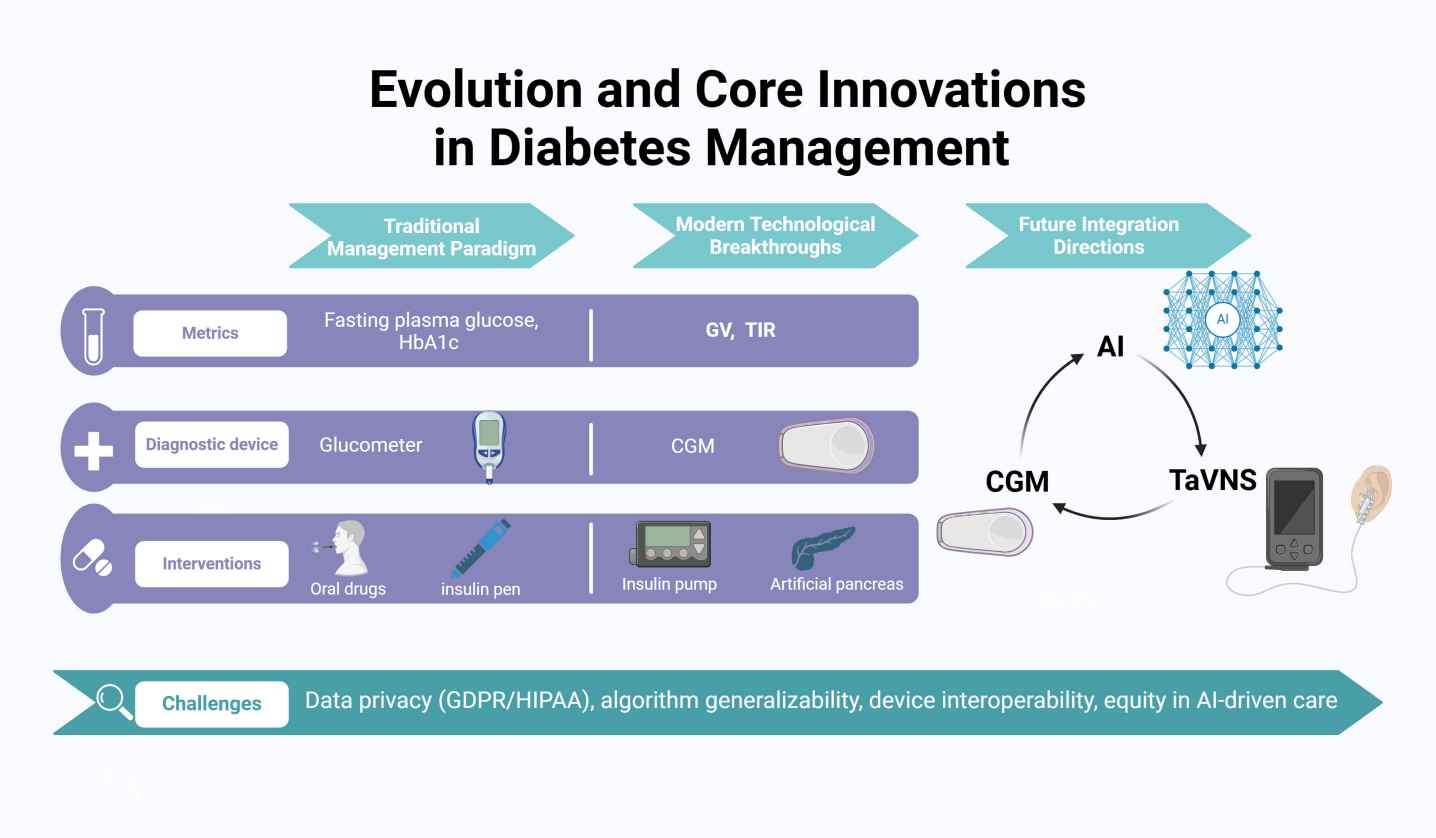
Evolution and core innovations in diabetes management. Evolution and core innovations in diabetes management such as the shift from traditional metrics (fasting plasma glucose, HbA1c) to modern measures (glucose variability, time in range), diagnostic tools (glucometers vs. continuous glucose monitoring), and interventions (oral drugs, insulin pens vs. insulin pumps, artificial pancreas systems). The integration of AI and taVNS with CGM represents a future direction in diabetes care. Additionally, it addresses challenges in AI-integrated diabetes care, such as data privacy (GDPR/HIPAA), algorithm generalizability, device interoperability, and equity in AI-driven care.

Diabetes remains a global health crisis, profoundly impacting morbidity, mortality, and healthcare expenditure. Under the Traditional Management Paradigm, fasting blood glucose and glycated hemoglobin (HbA1c, 1) served as cornerstone diagnostic metrics, with interventions typically relying on invasive fingerstick glucometers, oral hypoglycemic agents, and insulin pens. Modern Technological Breakthroughs have revolutionized care through continuous glucose monitoring (CGM), enabling data-driven optimization of glucose variability (GV, 2) and time-in-range (TIR, 3) as therapeutic targets. Innovations like insulin pumps and hybrid closed-loop artificial pancreas systems further exemplify this progress. Future Integration Directions lie in combining AI, CGM, and transcutaneous auricular vagus nerve stimulation (taVNS) – a promising non-pharmacological therapy representing a paradigm shift in diabetes management. This multimodal approach could enable real-time glucose-responsive neuromodulation while minimizing medication dependence. However, challenges remain, including the need for further clinical validation of taVNS and addressing data privacy and ethical issues related to AI in healthcare. **Figure 1** illustrates the evolution and core innovations in diabetes management. This paper examines these advancements, their transformative potential, and critical barriers to implementing next-generation diabetes care solutions.

## The evolving landscape of diabetes: epidemiology, burden, and the need for innovation

Diabetes mellitus has emerged as a significant global health concern, with Type 2 diabetes (T2D) accounting for the majority of cases. The prevalence of T2D has been rapidly increasing worldwide, particularly affecting younger age groups. Epidemiological studies have highlighted the substantial burden of diabetes, not only in terms of mortality but also in terms of morbidity and healthcare costs. One seminal study conducted in the United States estimated that diabetes accounted for 5.9% of deaths among adults aged 20 years and above, with T2D being the predominant contributor to this mortality rate. Globally, the number of deaths from T2D across all age groups has grown from 238,100 to 723,700 since 1990, a 203.9% increase (4). The global incidence rate of T2D in youth and young adults rose from 56.02 per 100,000 in 1990 to 123.86 per 100,000 in 2021 (5). Moreover, the burden of T2D tends to be higher in socioeconomically disadvantaged populations, exacerbating health disparities. This underscores the urgent need for targeted interventions to address the social determinants of health and mitigate the impact of diabetes on vulnerable communities (6). In addition to mortality, diabetes is associated with a spectrum of complications, including cardiovascular disease, renal failure, neuropathy, and retinopathy, which significantly reduce patients’ quality of life and impose substantial economic burdens on the healthcare system (7). Furthermore, the rising prevalence of prediabetes, a precursor to T2D, poses additional challenges. A study in China estimated that approximately 35.7% of adults had prediabetes, highlighting the need for early intervention strategies to prevent the progression to overt diabetes (8). Recent evidence also suggests an association between diabetes and an increased risk of cancer. A meta-analysis of global studies involving over 891,000 participants found that prediabetes was associated with a 15% higher risk of developing cancer. These findings underscore the interconnectedness of diabetes with other chronic diseases and emphasize the importance of adopting a holistic approach to diabetes management (9).

In diabetes care, traditional markers like HbA1c offer insights into average glycemic control but fail to capture short-term fluctuations in blood glucose levels, known as GV. GV refers to the extent of blood glucose fluctuations over time and has been linked to adverse outcomes, including microvascular and cardiovascular complications (10, 11). Advances in CGM have made it feasible to assess GV using metrics like the coefficient of variation (CV) and mean amplitude of glucose fluctuation. Clinically, a CV <36% indicates stable glucose levels, while higher values signal instability and predict severe hypoglycemic episodes within six months (10, 12). TIR, another key CGM metric, reflects the percentage of time glucose levels remain within a target range (typically 3.9–10.0 mmol/L). TIR provides a holistic view of glycemic control, encompassing both average glucose levels and variability. Studies have highlighted its clinical importance, with higher TIR correlating to fewer hypo- and hyperglycemic events, and reducing severe hypoglycemia risk by 46% (13, 14). Clinical guidelines recommend TIR levels exceeding 70%, adjusted for specific patient populations, making it a practical target for optimizing care.

Therefore, there is an urgent need for innovative solutions to address the challenges of diabetes. AI and personalized care are seen as key elements in the future management of diabetes. AI can analyze vast amounts of data to predict an individual’s risk of developing diabetes and formulate personalized treatment plans (15). Moreover, personalized care can provide tailored treatment and management strategies based on each patient’s specific circumstances.

## Technological advancements in glucose monitoring: enabling personalized diabetes management

Advances in blood glucose monitoring technologies have revolutionized diabetes management, enabling more personalized and effective glycemic control strategies. CGM systems have become central to modern care, providing real-time glucose data and trends. Studies consistently show that CGM use improves glycemic control, reduces hypoglycemia, and enhances patient satisfaction compared to traditional self-monitoring methods (13, 16).

The integration of CGM with insulin pumps has led to closed-loop systems, or artificial pancreas systems, which automate insulin delivery based on glucose feedback. These systems mimic pancreatic function, offering tailored insulin delivery that significantly increases time spent within target glucose ranges and reduces hypoglycemia (14, 17). The hybrid closed-loop system further enhances flexibility, allowing manual adjustment of meal timing while maintaining automated basal insulin delivery, thus providing greater customization to achieve further personalized care (18).

Emerging technologies are set to improve glucose monitoring further. Implantable sensors provide long-term monitoring without frequent insertions, boosting adherence and comfort. Non-invasive devices eliminate the need for painful fingersticks, making monitoring more user-friendly (19, 20). Smartphone apps with glucose-tracking features empower patients to analyze and share data, fostering active disease management (21).

The convergence of CGM with artificial intelligence is accelerating precision medicine applications. The Dexcom G7 system synchronizes CGM data directly to Apple Health via Bluetooth, allowing real-time glucose trend visualization on iPhones or Apple Watches. Its cloud-based API supports third-party applications in generating personalized dietary recommendations. Currently, the accuracy and safety of Dexcom G7 are verified (22). In a large-scale real-world study of adults with type 2 diabetes without insulin and poor glycemic control, the use of Dexcom CGM was associated with significant improvements in glycemic control within 12 months. The use of the high alarm system function was positively correlated with blood glucose outcome. The high proportion of CGM use within 12 months indicates that continued CGM use is beneficial in this population (23). A clinical trial in Rwanda with type 1 diabetes using Dexcom caused a significant increase in target glucose duration and a decrease in HbA1c (24). Such integrations exemplify the transition from passive monitoring to proactive, data-driven care models. The ongoing advancement of AI technologies has significantly enhanced cost-effectiveness in healthcare, particularly demonstrating substantial potential in diabetes care. In a Chinese study involving 251,535 participants for diabetic retinopathy (DR) screening, AI systems demonstrated cost-effectiveness in most scenarios, though outcomes may be influenced by screening sensitivity parameters (25). A Japanese modeling study revealed that implementing AI-powered DR screening for diabetes management yields superior cost-effectiveness compared to conventional approaches (26). Research from Australia also confirmed that the deployment of AI-assisted DR screening in primary care settings achieved both operational efficiency and cost savings across Indigenous and non-Indigenous populations (27). These collective findings confirm that progress in AI technology continues to optimize healthcare cost-benefit ratios, establishing a robust foundation for scalable applications in diabetes treatment.

These technological strides collectively empower patients and clinicians to implement truly personalized diabetes management strategies, fundamentally transforming the care continuum from episodic intervention to continuous health optimization.

## TaVNS: a multi-target approach to diabetes management

TaVNS, a non-invasive neuromodulation technique, specifically targets auricular regions—the only areas of the ear with vagus nerve distribution (28). As a crucial pathway connecting the central and peripheral nervous systems, the auricular branch of the vagus nerve, when activated, transmits electrical signals to the nucleus tractus solitarius (NTS), which subsequently modulates brain activity and peripheral organ functions bidirectionally (29). This mechanism enhances parasympathetic activity and restores autonomic balance (30), providing a neuroscientific basis for metabolic regulation.

Recent studies further elucidate the potential mechanisms of taVNS in diabetes management. For instance, taVNS has been shown to modulate blood glucose in Zucker diabetic fatty (ZDF) rats by regulating intestinal melatonin receptors and melatonin secretion, suggesting a novel pathway for glycemic control (31) Additionally, taVNS improved depressive-like behaviors in ZDF rats via the P2X7R/NLRP3/IL-1β pathway, highlighting its potential in addressing diabetes-related comorbidities (32) Furthermore, taVNS has been found to enhance insulin receptor expression in the liver, skeletal muscle, and pancreas, potentially improving insulin resistance (33). Beyond diabetes, taVNS has shown promise in treating polycystic ovary syndrome (PCOS), a condition often associated with insulin resistance and metabolic dysfunction, by modulating autonomic nervous system activity and restoring metabolic homeostasis (34).

Clinical research supports its benefits in patients with impaired glucose tolerance (35). While direct diabetes evidence requires validation, taVNS demonstrates multi-target mechanisms across diseases. A 171-patient trial showed taVNS reduced stress in tinnitus patients and enhanced parasympathetic function in 80% of participants (36). By restoring autonomic balance, taVNS regulates insulin secretion to lower blood glucose (37). Notably, sepsis patients exhibited reduced pro-inflammatory cytokine and elevated anti-inflammatory markers post-taVNS (38), directly addressing diabetes-related chronic inflammation. Appetite modulation studies revealed taVNS lowers post-meal ghrelin (39), aiding weight control in obese T2D patients. Gastrointestinal benefits include improved gastric accommodation in functional dyspepsia (40) and constipation relief in constipation-predominant irritable bowel syndrome via immune modulation (41). These findings suggest taVNS may indirectly influence glucose metabolism by regulating gut hormone secretion and gut-brain axis communication.

Comorbidity management amplifies metabolic benefits. Four-week taVNS improved sleep and reduced anxiety in healthcare workers (42), crucial as poor sleep (43) and anxiety (44) impair glycemic control. Pain relief effects—reducing joint inflammation in hand osteoarthritis (45) and chemotherapy-induced neuropathy (38)—may lessen the stress-induced glucose peak caused by chronic pain in diabetics.

In summary, taVNS offers multi-modal action: autonomic regulation, anti-inflammation, appetite/gut modulation, and comorbidity management. These mechanisms provide theoretical and practical foundations for diabetes intervention. Though large-scale trials are needed, cross-disease evidence highlights its therapeutic potential in comprehensive diabetes care.

## Integrating taVNS with AI: a paradigm shift in diabetes care

Artificial pancreas systems, which integrate CGM with automated insulin delivery, have shown significant efficacy in maintaining glycemic control by dynamically adjusting insulin doses based on real-time glucose data. Building upon this framework, the incorporation of taVNS offers a complementary approach to further enhance glucose regulation through noninvasive neuromodulation.

Emerging evidence supports CGM as a transformative tool for non-insulin-treated T2D. Clinical studies demonstrate CGM’s association with improved glycemic control (46), reduced hypoglycemia risk, and enhanced healthcare efficiency (47). An 182-patient randomized controlled trial confirmed glycemic improvements in non-insulin users (48), while real-world data from 70 million patients revealed sustained benefits across therapies (47). Integration with AI-powered digital therapeutics further amplifies outcomes: a trial involving 118 non-insulin-treated T2D patients showed dual improvements in glucose regulation and weight management (49). CGM data also addresses clinical challenges in patients with discordant HbA1c and average glucose levels through patient-specific predictive models (50). These advancements position CGM-AI integration as a cornerstone for next-generation diabetes management, extending beyond traditional insulin-centric paradigms.

The proposed AI-taVNS-CGM system operates through a closed-loop feedback mechanism. Real-time CGM data is processed by a reinforcement learning (RL) algorithm, such as the RL-DITR system (51). This algorithm learns the optimal insulin regimen by analyzing glycemic state rewards through patient model interactions, aiming to control blood glucose levels and reduce hypoglycemia and hyperglycemia-ketotic events. Based on this model, suitable taVNS parameters can be trained. CGMfroster based on Transformer architecture, through self-supervised learning from large-scale CGM data learning individual blood glucose dynamic characteristics, can accurately characterization of individual fasting glucose homeostasis maintenance and postprandial hyperglycemia adaptation dynamic behavior, can assist the diagnosis, judgment and complication prediction, type 2 diabetes into diabetic subtype, accurately predict postprandial blood glucose response, and according to the prediction results for diabetes patients with personalized dietary advice, lifestyle intervention recommended (52).

**Figure 2:**
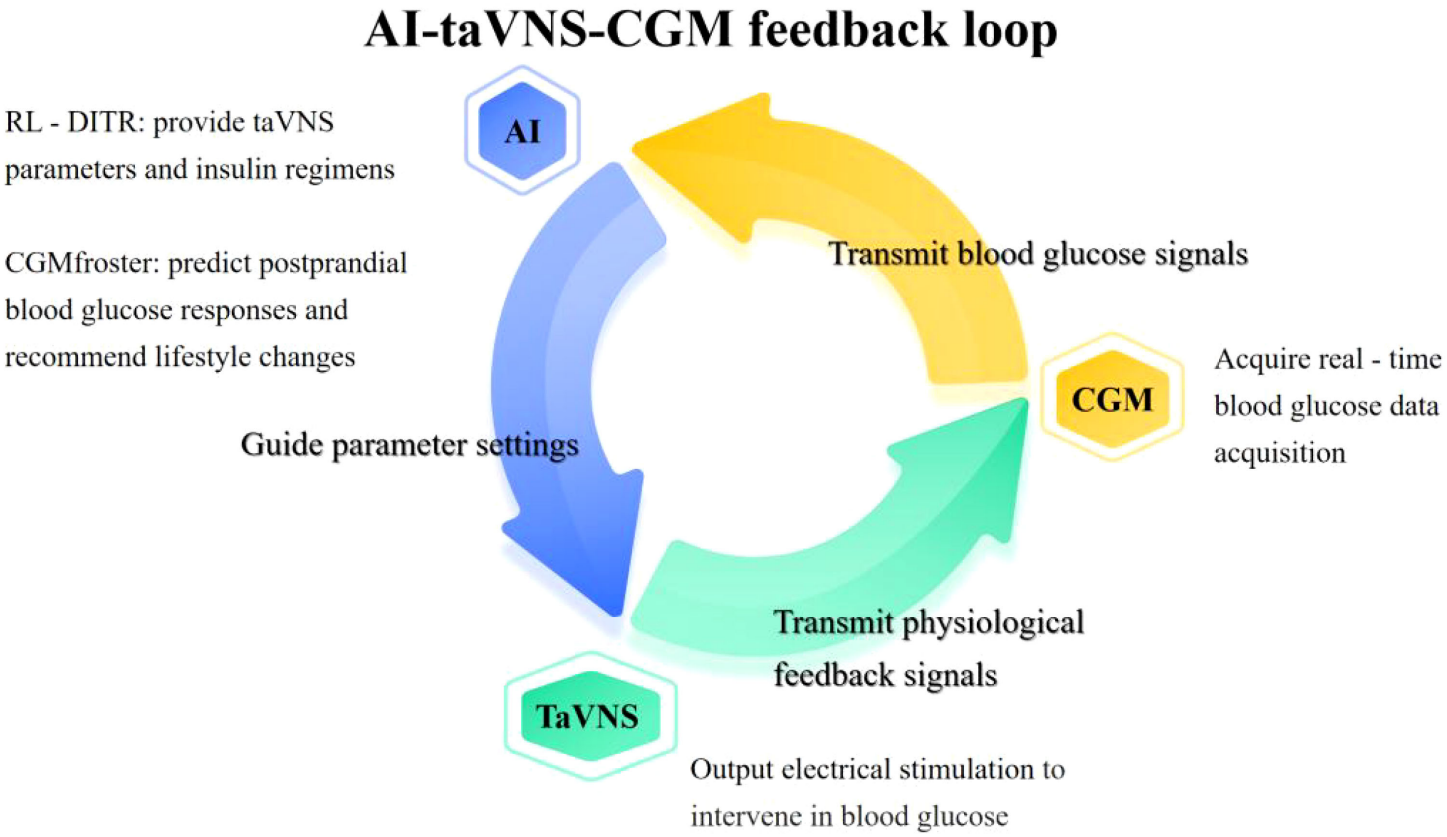
AI-taVNS-CGM feedback loop. This figure illustrates the dynamic interaction between AI, taVNS, and CGM in diabetes management. It shows how AI processes data from CGM to dynamically adjust taVNS parameters (e.g., stimulation frequency/duration) in response to real-time blood glucose data. The loop also demonstrates how taVNS provides physiological feedback signals that are fed back into the AI system to further refine the treatment regimen. This integrated system enables personalized, real-time diabetes management by combining the strengths of AI-driven data analysis, CGM’s continuous glucose monitoring, and taVNS’s non-invasive neuromodulation.

In this context, taVNS can be integrated with CGM and AI algorithms to provide a non-invasive, personalized approach to glycemic management for individuals not dependent on insulin pumps. By receiving real-time CGM data via smartphones or dedicated software and incorporating user inputs on meal intake and physical activity, the system can analyze these inputs to determine optimal taVNS stimulation frequency and duration. This approach leverages the benefits of taVNS for a broader patient population, offering a novel, non-pharmacological intervention for glycemic control. **Figure 2** illustrates the AI-taVNS-CGM feedback loop.

## Empowering patients through AI-driven self-management and community engagement

Patient self-management plays a crucial role in diabetes care, empowering individuals to take an active role in monitoring their blood glucose levels, adhering to treatment regimens, and making lifestyle modifications. Structured self-management education programs, often delivered by healthcare providers or certified diabetes educators, help patients develop essential skills for managing their condition effectively (53). These programs typically cover glucose monitoring, medication management, healthy eating, physical activity, and coping strategies for dealing with diabetes-related challenges. Studies have demonstrated that patients who actively engage in self-management experience better glycemic control, reduced risk of complications, and improved quality of life (54).

Community health education programs aim to raise awareness about diabetes risk factors, prevention strategies, and management techniques among at-risk populations. These initiatives utilize various platforms, including workshops, seminars, social media, and community health fairs, to disseminate information and promote healthy lifestyle behaviors. Research has shown that culturally tailored educational interventions are particularly effective in engaging diverse communities and fostering behavior change (55). Moreover, community-based programs provide opportunities for peer support, enabling individuals with diabetes to share experiences, seek advice, and access resources in a supportive environment (56).

To further enhance patient engagement, innovative strategies such as gamified mobile applications have been developed to make diabetes management more interactive and enjoyable. These apps use game-like elements, such as rewards, challenges, and progress tracking, to motivate patients to adhere to their treatment plans and maintain healthy habits. For instance, mySugr allows users to log blood glucose levels, track meals, and earn points for consistent self-monitoring, which can be redeemed for virtual or real-world rewards. Such gamification techniques have been shown to improve glycemic control in people with type 1 and type 2 diabetes (57). Crucially, integrating patient-reported outcomes (PROs) into AI systems enables dynamic intervention customization. This synergy extends to device optimization: community-generated data from CGM and taVNS devices can be aggregated on cloud platforms, where AI algorithms refine device parameter based on population-level insights. These innovations establish a self-reinforcing cycle: engaged patients produce richer datasets, enhancing AI precision and enabling personalized device adjustments, which in turn improve treatment adherence and outcomes.

By actively participating in community programs, patients not only gain access to valuable resources and peer support but also contribute to a richer data ecosystem that enhances AI-driven diabetes care. Notably, diabetes patient communities are not just beneficiaries of technological innovations but vital contributors to the innovation process. Their real-world usage experiences and demand feedback play a crucial role in emerging enterprises’ product design iterations, driving the development of more human-centric and efficient diabetes management solutions (58). AI systems, leveraging community-generated data, can provide personalized guidance tailored to individual needs, such as dietary recommendations, exercise plans, and medication adjustments. Furthermore, community-based supervision fosters accountability, encouraging patients to adhere to their treatment regimens and adopt healthier behaviors. This collaborative approach—combining patient engagement, community support, and AI-driven insights—creates a sustainable feedback loop that improves both individual outcomes and population-level diabetes management.

The integration of community engagement and AI-driven data ecosystems creates a transformative framework for diabetes management. By embedding patients within community networks, AI systems gain structured access to longitudinal health data from wearable devices and patient-reported outcomes through centralized platforms (59). This data synergy enables machine learning models to generate personalized glycemic control recommendations, such as adaptive insulin dosing algorithms and neuromodulation parameter adjustments tailored to individual metabolic patterns.

## Future directions: overcoming challenges in AI-taVNS integration for scalable diabetes care

The application of taVNS in diabetes management remains in the mechanistic exploration stage. Current evidence is primarily derived from preclinical studies and small-scale clinical trials, with limited data on its efficacy, safety, and long-term outcomes in diverse diabetic populations. While taVNS has demonstrated glycemic benefits in ZDF rats and patients with impaired glucose tolerance, its effects in individuals with established type 1 or type 2 diabetes require further validation. Future research should focus on large-scale, randomized controlled trials to evaluate taVNS’s safety, optimal stimulation parameters, long-term adherence, and cost-effectiveness compared to conventional therapies. Additionally, studies should explore its potential synergies with existing treatments, such as CGM and AI-driven interventions, to develop comprehensive, personalized diabetes care strategies. Despite these challenges, taVNS holds significant promise as a non-invasive, neuromodulatory approach to diabetes management, particularly for patients with comorbid conditions such as depression or PCOS. Its potential to address both metabolic and psychological aspects of diabetes underscores the need for continued investigation into its mechanisms and clinical applications. Unlike pharmacological interventions requiring hepatic metabolism and carrying drug interaction risks, taVNS delivers targeted neuromodulation through wearable stimulators. This electrophysiological mechanism circumvents systemic toxicity while enabling real-time AI-powered parameter adjustments—a key advantage for personalized chronic disease management (60). Notably, taVNS demonstrates therapeutic potential for diabetes comorbidities like depression (61) and polycystic ovary syndrome, potentially reducing polypharmacy burdens through its neuromodulatory effects on neuroendocrine-immune pathways.

AI-CGM-taVNS systems face ethical and technical challenges in diabetes management. Data privacy concerns arise from third-party access to cloud-based CGM metrics for AI-driven taVNS optimization, requiring strict GDPR/HIPAA compliance (62). To mitigate these biases, federated learning has emerged as a key solution, enabling institutions to collaboratively train shared models without exchanging raw data (63). This decentralized approach preserves patient privacy, complies with data protection regulations, and reduces algorithmic bias by incorporating diverse datasets. Additionally, adversarial debiasing techniques can be employed to further minimize demographic disparities in model performance (64).

Technical hurdles include device interoperability gaps. Marfoglia et al. transformed clinical data into the Fast Healthcare Interoperability Resources (FHIR) model, enhancing semantic interoperability and facilitating the reuse of real-world data (65). Salgado-Baez et al. proposed a FHIR-based standard solution for the comprehensive analysis of drug data in a large German hospital, successfully achieving drug order message generation and ensuring drug interaction and standardized data exchange (66). Similarly, IEEE P1752 (67) offers standardized semantics for mobile health data, facilitating meaningful description, exchange, and analysis of sleep and physical activity metrics, which are critical for diabetes management. These frameworks enhance interoperability and ensure data clarity for clinical/research applications.

In low-resource settings, AI-driven tools hold transformative potential but require balanced implementation. Wong et al. outline four pillars: infrastructure, data management, education, and ethical AI practices, emphasizing localized data collection (68). Evidence from India highlights AI’s role in improving healthcare accessibility, such as automated diagnostics reducing reliance on scarce expertise (69, 70). However, systemic risks persist, including biased datasets that disproportionately harm marginalized populations (71, 72) and digital illiteracy barriers (73). To address these limitations, integrated strategies should combine lightweight AI systems for low-bandwidth environments, policy reforms fostering public-private partnerships, and tiered training programs enhancing both clinician expertise and community digital literacy. Crucially, AI-taVNS integration must embed social support, such as infrastructure upgrades and culturally adapted health education, to avoid oversimplifying chronic disease management (74).

Clinical adoption of AI-CGM-taVNS systems faces cost barriers in resource-limited settings and demands clinician/patient training for AI-driven insights. Future priorities include interdisciplinary consortia for open-source AI architectures, randomized trials validating algorithm-driven taVNS protocols, and regulatory frameworks for AI-medical device integration. Collaborative efforts leveraging federated learning, FHIR/IEEE P1752 interoperability standards, and cost optimization can ensure equitable access to AI-enhanced diabetes care across diverse populations.

## Conclusion

The evolving landscape of diabetes management is increasingly driven by innovative approaches that address the growing global burden of diabetes. Traditional metrics like HbA1c are insufficient in capturing the complexity of glycemic control, highlighting the need for more comprehensive measures such as GV and TIR (75). These metrics, supported by CGM technology, provide real-time data that empowers both patients and healthcare providers to optimize diabetes management. AI algorithms further enhance this process by predicting risks and personalizing treatment plans, addressing both short-term fluctuations and long-term stability.

Empowering patients through AI-driven self-management tools and community engagement is crucial for sustainable improvements. Gamified apps and social media communities enhance adherence and provide peer support, while integrating PROs with AI enables dynamic, personalized interventions.

However, challenges remain in the practical application of AI and taVNS integration, including data privacy concerns, algorithmic bias, device interoperability, and equity in AI-driven care. Future research should focus on large-scale clinical trials, cost-effectiveness analyses, and interdisciplinary collaboration. Policy support, open data protocols, and regulatory frameworks are essential for widespread adoption. Implementation strategies should combine resource-efficient computational tools for diverse healthcare settings, policy incentives for cross-sector technology co-development, and scalable education initiatives addressing both clinical AI adoption and community health literacy. By leveraging these advancements, we can tailor interventions to individual needs, optimize glycemic control, and improve the quality of life for patients with diabetes.

## Data Availability

The original contributions presented in the study are included in the article/supplementary material. Further inquiries can be directed to the corresponding author/s.
